# Whole-Genome Sequencing of Clinical Isolates of Mycobacterium tuberculosis Isolated before and after Treatment

**DOI:** 10.1128/mra.01336-22

**Published:** 2023-01-18

**Authors:** Eon-Min Ko, Hyungjun Kim, Ji-A Jeong, Sungkyoung Lee, Seonghan Kim

**Affiliations:** a Division of Bacterial Disease Research, Center for Infectious Disease Research, National Institute of Infectious Diseases, National Institute of Health, Korea Disease Control and Prevention Agency, Osong, Republic of Korea; b Division of Infectious Disease Control, Bureau of Infectious Disease Policy, Korea Disease Control and Prevention Agency, Osong, Republic of Korea; University of Rochester School of Medicine and Dentistry

## Abstract

Mycobacterium tuberculosis clinical isolates CN177_0W, CN177_2W, CB060_0W, and CB060_2W were isolated from two tuberculosis patients in South Korea. Here, we report the whole-genome sequences of clinical isolates of M. tuberculosis isolated before and after tuberculosis treatment.

## ANNOUNCEMENT

Tuberculosis is an infectious disease caused by Mycobacterium tuberculosis and one of the leading causes of death worldwide. Tuberculosis treatment requires an appropriate combination of drugs, and the number of viable bacilli in the sputum usually decreases significantly during the first 2 weeks after treatment initiation (1). The presence of viable bacilli in the sputum of tuberculosis patients for more than 2 weeks after treatment suggests that they are still infectious and require prolonged treatment (2, 3). We performed whole-genome sequencing of isolates of M. tuberculosis isolated before treatment initiation and after 2 weeks of treatment to confirm the genetic changes associated with the treatment.

Here, we present the genome sequences of four M. tuberculosis clinical isolates from two patients before standard regimen initiation and 2 weeks after treatment. Study participants were recruited from a tuberculosis cohort from 2016 to 2018. The Institutional Review Board of the Korea Disease Control and Prevention Agency approved this study (2019-04-05-2C-A). Four clinical isolates of M. tuberculosis (CN177_0W, CN177_2W, CB060_0W, and CB060_2W) were isolated from the sputum of tuberculosis patients at Chungnam National University Hospital (CN177_0W and CN177_2W) and Chungbuk National University Hospital (CB060_0W and CB060_2W) in South Korea. Clinical isolates were isolated from the sputum samples using both Lowenstein-Jensen (L-J) medium and a mycobacterial growth indicator tube (MGIT) culture system. Staining of acid-fast bacilli (AFB) with Ziehl-Neelsen stain and MPT64 antigen detection using Bioline TB Ag MPT64 test (Abbott, Chicago, IL) were used to confirm the positive culture isolates. AFB staining- and MPT64 detection-negative samples were assessed by PCR using the PowerChek MTB/NTM real-time PCR kit (Kogene Biotech, Seoul, Korea).

Genomic DNA was isolated using the Quick-DNA fungal/bacterial miniprep kit (Zymo Research, Irvine, CA) following the manufacturer’s instructions. The TruSeq Nano DNA kit (Illumina, San Diego, CA) was used to generate paired-end (2 × 150-bp) libraries. Whole-genome sequencing of the four libraries was conducted on an Illumina NovaSeq 6000 platform. Reads generated through sequencing were quality validated and trimmed using Prinseq (v0.20.4) (4). After filtering, totals of 1,922,648, 1,791,360, 1,894,722, and 2,225,060 reads were generated by sequencing CN177_0W, CN177_2W, CB060_0W, and CB060_2W, respectively. The reads were assembled *de novo* using SPAdes (v3.15.2), resulting in 65 to 74 contigs with *N*_50_ values of ~136,458 to 151,906 (5). Genome annotation was performed using Prokka (v1.14.6) (6). Default parameters were used for all software. Genome assembly statistics and annotation features are shown in Table 1.

**TABLE 1:** Assembly statistics of whole-genome sequencing

Characteristic	CN177_0W	CN177_2W	CB060_0W	CB060_2W
Total no. of reads (bp)	1,922,648	1,791,360	1,894,722	2,225,060
Genome size (bp)	4,370,051	4,370,780	4,369,234	4,368,804
No. of contigs	74	74	65	71
GC content (%)	65.6	65.6	65.6	65.6
Coverage (×)	126	118	124	146
*N*_50_ value (bp)	137,416	151,906	151,903	136,458
No. of coding sequences	4,039	4,044	4,036	4,032
No. of rRNAs	3	3	3	3
No. of tRNAs	52	52	52	52
No. of transfer-messenger RNAs	1	1	1	1
GenBank accession no.	JAOUST000000000	JAOUSS000000000	JAOUSR000000000	JAOUSQ000000000
BioSample accession no.	SAMN31178387	SAMN31178388	SAMN31178389	SAMN31178390
SRA accession no.	SRR21820127	SRR21820126	SRR21820125	SRR21820124

### Data availability.

Accession numbers are available in Table 1.
